# Selective Transgenic Expression of Mutant Ubiquitin in Purkinje Cell Stripes in the Cerebellum

**DOI:** 10.1007/s12311-016-0838-1

**Published:** 2016-12-13

**Authors:** Bert M. Verheijen, Romina J. G. Gentier, Denise J. H. P. Hermes, Fred W. van Leeuwen, David A. Hopkins

**Affiliations:** 1 Department of Neuroscience, Faculty of Health, Medicine and Life Sciences, Maastricht University, Maastricht, The Netherlands; 2 Lab of Experimental Neurology, Brain Center Rudolf Magnus, University Medical Center Utrecht, Utrecht, The Netherlands; 3 Department of Medical Neuroscience, Dalhousie University, Halifax, Nova Scotia Canada

**Keywords:** Ubiquitin-proteasome system, Ubiquitin-B^+1^, Purkinje cell stripes, Zebrin II, Heat shock protein 25, Cerebellum

## Abstract

The ubiquitin-proteasome system (UPS) is one of the major mechanisms for protein breakdown in cells, targeting proteins for degradation by enzymatically conjugating them to ubiquitin molecules. Intracellular accumulation of ubiquitin-B^+1^ (UBB^+1^), a frameshift mutant of ubiquitin-B, is indicative of a dysfunctional UPS and has been implicated in several disorders, including neurodegenerative disease. UBB^+1^-expressing transgenic mice display widespread labeling for UBB^+1^ in brain and exhibit behavioral deficits. Here, we show that UBB^+1^ is specifically expressed in a subset of parasagittal stripes of Purkinje cells in the cerebellar cortex of a UBB^+1^-expressing mouse model. This expression pattern is reminiscent of that of the constitutively expressed Purkinje cell antigen HSP25, a small heat shock protein with neuroprotective properties.

## Introduction

Efficient protein quality control is essential for the maintenance of cellular homeostasis to prevent accumulation of damaged and toxic proteins that would be detrimental to cells and their function. The ubiquitin-proteasome system (UPS) is one of the major mechanisms for targeted protein breakdown in cells, tagging proteins for degradation by enzymatically conjugating them to ubiquitin molecules [1, 2]. Impaired protein quality control and degradation are often associated with aging and disease [3, 4].

Ubiquitin-B^+1^ (UBB^+1^) is a frameshift mutant of ubiquitin-B (UBB) that has been found to accumulate in a variety of disorders, including neurodegenerative diseases [5, 6]. UBB^+1^ is thought to arise through “molecular misreading,” a process that introduces mutations not present in genomic DNA into repeating motifs (e.g., GAGAG motifs) of mRNA resulting in mutant proteins [5, 7]. UBB^+1^ lacks a C-terminal glycine residue and therefore cannot ubiquitinate other proteins, but can still be ubiquitinated itself. Low levels of UBB^+1^ are efficiently degraded by the proteasome via the ubiquitin-fusion degradation (UFD) pathway [8]. At high concentrations, however, UBB^+1^ is a potent inhibitor of the UPS [9, 10]. Interestingly, studies in yeast have indicated that UBB^+1^ is an inhibitor of deubiquitinating enzymes (DUBs) [11]. In addition, UBB^+1^ causes neuritic beading of mitochondria in association with neuronal degeneration [12]. This suggested an effect of UBB^+1^ on mitochondrial function. It was recently reported that accumulation of basic amino acids at mitochondria dictates the cytotoxicity of UBB^+1^ [13].

To study the effects of UBB^+1^ accumulation in vivo, UBB^+1^-expressing mouse models have been generated. Transgenic mice overexpressing human UBB^+1^ in brain show increased levels of ubiquitinated proteins in the forebrain and display behavioral deficits (e.g., impaired contextual memory) that are compatible with neurodegenerative disease [14].

Crossbreeding UBB^+1^ mice to relevant disease models has shown disease-modifying effects [15, 16]. A comprehensive phenotypic screening of UBB^+1^-expressing mice revealed a respiratory phenotype [17]. Concordantly, expression of UBB^+1^ was found in brainstem nuclei involved in respiratory control. UBB^+1^ immunoreactivity in Alzheimer’s disease (AD) patients was seen in similar areas in the brainstem, suggesting a link between neuropathology in these brainstem areas and the respiratory and swallowing dysfunctions that are often seen in AD patients [17].

In the present study, we show that UBB^+1^ is specifically expressed in a subset of parasagittal stripes of Purkinje cells (PCs) in the cerebellar cortex of a UBB^+1^-expressing mouse model. This expression pattern is similar to that of the constitutively expressed PC antigen HSP25.

## Materials and Methods

### Animals

UBB^+1^-expressing transgenic mice (line 3413, JAX C57BL/6-Tg(Camk2a-UBB)3413-1Fwvl/J) were described previously [14]. Male mice (*n* = 14; eight 3-month-old mice, two 7-month-old mice, and four 15-month-old mice) were kept under standard animal housing conditions: a 12/12 h light-dark cycle with food and water ad libitum in specific pathogen-free conditions. Non-transgenic littermates were used as controls. All animal experiments were performed according to national animal welfare law and under guidance of the animal welfare committees of the Royal Netherlands Academy of Arts and Sciences (KNAW) and of Maastricht University.

### Tissue Processing and Immunohistochemistry

Adult male mice were deeply anesthetized using sodium pentobarbital and were transcardially perfused with 0.9% NaCl, followed by 4% paraformaldehyde in phosphate-buffered saline (PBS) (pH 7.4). After removal, the brains were fixed overnight in 0.1 M phosphate buffer containing 4% paraformaldehyde (pH 7.4). The brains were subsequently stored in 1% sodium azide (NaN_3_) in PBS at 4 °C until further processing. All brains were embedded in gelatin and sectioned on a Vibratome (Leica VT 1200S, Wetzlar, Germany) into 50 μm thick coronal or sagittal sections.

For immunohistochemistry, sections were incubated with primary antibodies at 4 °C overnight. Primary antibodies included the following: polyclonal rabbit anti-mouse UBB^+1^ (Ubi3, 1:1000, Dr. F.W. van Leeuwen, bleed date 16/09/97), monoclonal mouse anti-zebrin II/aldolase C (1:100, Dr. R. Hawkes, Calgary), monoclonal mouse anti-calbindin-D28k (1:25,000, Swant), polyclonal rabbit anti-HSP25 (1:1000, Enzo Life Sciences), and monoclonal mouse anti-HSP25 (p-HSP27 (B3), 1:400, Santa Cruz Biotechnology). Antibodies were diluted in Tris-buffered saline (TBS) containing 0.5% Triton X-100 (pH 7.6). After incubation with primary antibodies, sections were rinsed in TBS and incubated with biotinylated donkey anti-rabbit or anti-mouse antibodies (1:400) (Jackson Laboratories) followed by avidin-biotin-peroxidase (ABC) kit (Vector) at RT for 1 h. The staining was visualized with 3,3′-diaminobenzidine (DAB) tetrahydrochloride intensified by 0.2% nickel ammonium sulfate (pH 7.6). The sections were mounted on gelatin-coated glass slides, air-dried, dehydrated, and coverslipped using Pertex (Histolab). For immunofluorescence experiments, secondary antibodies with a fluorescent tag (donkey anti-mouse/rabbit Alexa 488/594) were used. Images were made using an Olympus BX51 microscope connected to a digital camera or a whole-slide scanning system (.slide, Olympus).

## Results and Discussion

**Fig. 1:**
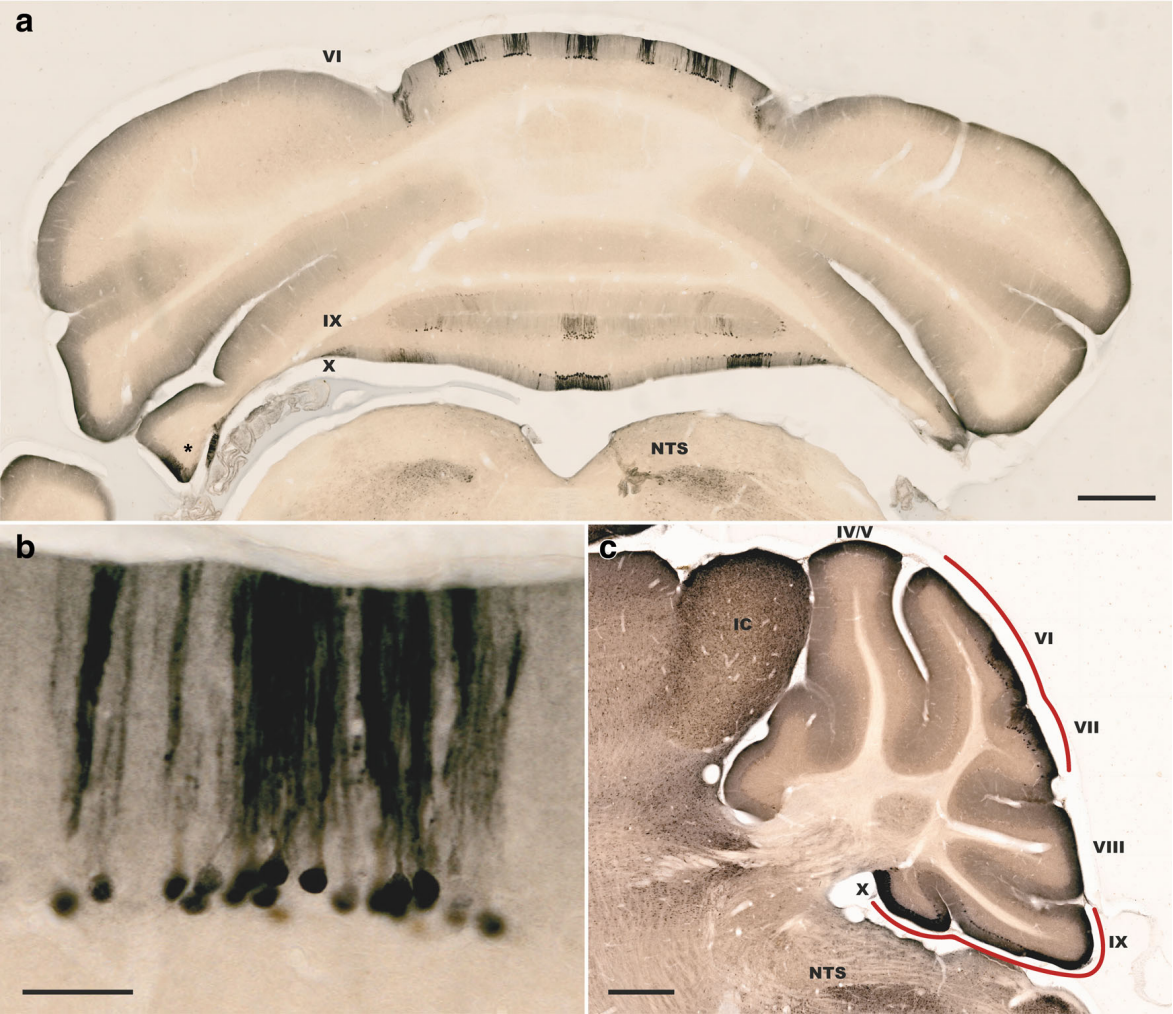
Expression pattern of mutant ubiquitin (UBB^+1^) in the mouse cerebellum. Distribution of UBB^+1^ in the cerebellum of a UBB^+1^-expressing transgenic mouse line. A coronal section reveals restricted expression of UBB^+1^ in parasagittal stripes of Purkinje cells (*PCs*) in the vermis of the cerebellar cortex (**a** and **b**). A sagittal overview shows expression of UBB^+1^ in lobules VI, VII, IX, and X (**c**). *IC* inferior colliculus, *NTS* nucleus of the solitary tract. *Asterisk* denotes additional UBB^+1^ expression in the cerebellar hemisphere. *Scale bars* **a** and **c** 500 μm, **b** 50 μm

**Fig. 2:**
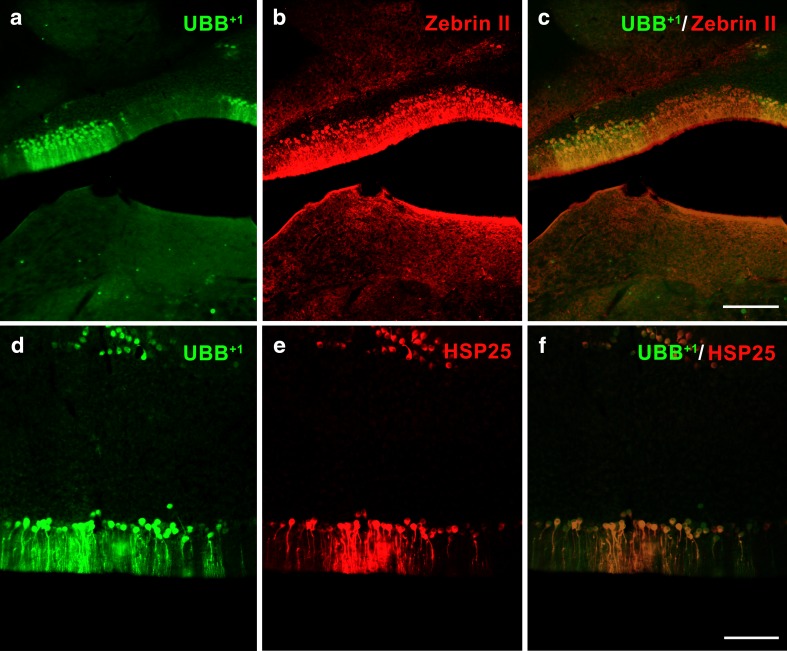
Co-expression of mutant ubiquitin (UBB^+1^) and HSP25 in Purkinje cell (*PC*) stripes. UBB^+1^ is expressed in a subset of zebrin II-positive PC stripes in the cerebellum of a UBB^+1^-expressing mouse line (**a–c**). Co-immunofluorescence (**d–f**) shows co-expression of UBB^+1^ and HSP25. All sections are in the coronal plane (lobules IX–X). *Scale bars* **a–c** 200 μm, **d–f** 100μm

Transgenic mice expressing UBB^+1^ show a widespread distribution of UBB^+1^-positive neurons in the forebrain (e.g., cerebral cortex, striatum, hippocampal formation, and thalamus) as well as in the brainstem [17, 18]. Here, we demonstrate that UBB^+1^ is also expressed in the cerebellum of a UBB^+1^-expressing mouse model. PC expression of the transgene is expected based on the use of the Camk2a gene promoter, a gene of well-known functional significance in these cells. Immunohistochemistry reveals that UBB^+1^-positive cerebellar PCs are always located within parasagittal stripes in cerebellar lobules VI, VII, IX, and X as well as in the flocculus and paraflocculus (Fig. 1). This pattern shows a striking resemblance to a previously identified marker for PC stripes, the small heat shock protein HSP25 (HSP27, HSPB1) [19]. The cerebellum is highly compartmentalized into bilaterally symmetric anatomical and functional clusters, which can be visualized through the expression of certain molecular markers [20, 21]. HSP25 is constitutively expressed in the central nervous system of rodents, notably in the cerebellum, brainstem, hypothalamus, and spinal cord [19, 22, 23]. HSP25 is known to act as a molecular chaperone and has been specifically associated with the functioning of the UPS. For example, HSP25 has been proposed to confer resistance to proteasome inhibition in astrocytes [24]. HSP25 also possesses neuroprotective properties; HSP25-positive PCs appear to be more resistant to cell death than HSP25-negative PCs, and HSP25 is highly stress-inducible throughout the nervous system [25, 26]. Furthermore, differences in the expression of HSP25 have been reported in mutant mice and under the influence of genes controlling development [27, 28]. Further examination revealed that UBB^+1^-immunoreactive Purkinje neurons co-express HSP25 (Fig. 2). UBB^+1^ is absent in all brain regions of control mice [29].

UBB^+1^ was also detected in cerebellar PC stripes in another UBB^+1^-expressing transgenic mouse line [30] (line 6663, unpublished observations). UBB^+1^-expressing transgenic mice do not display behavioral deficits in motor coordination tasks [17, 29] and no (focal) loss of PCs was observed (calbindin-D28K immunostaining). Other changes in PC layer cytoarchitecture (e.g., dendrite complexity, synaptic connectivity, and structural plasticity) were not evaluated. Extensive molecular profiling of different PC clusters may provide new insights into important aspects of the fundamental anatomical organization of the cerebellum. HSP25-immunoreactive PCs are known to specifically express other markers, represent clusters that also include other cell types, and are associated with somatostatin 28-immunoreactive mossy fiber pathways [31, 32]. These insights might also reveal several aspects of development and differential vulnerability of PC clusters and neural circuits in the cerebellum [33].

The 3413 transgenic line may be of great interest in the future for dissecting functional contributions of the cerebellum, PCs, and possibly even sagittal zones and stripes, in neurodegenerative diseases such as AD. Diffuse senile plaques occur commonly in the cerebellum of AD patients and certain patients present with cerebellar ataxias [34–37]. Some studies propose that PCs are key players in these AD-associated cerebellar defects [38, 39]. Currently, there is no evidence of PC loss or axonal degeneration in these transgenic mice, but this should be examined in more detail in the future.
